# Electrospun Membranes as a Porous Barrier for Molecular Transport: Membrane Characterization and Release Assessment

**DOI:** 10.3390/pharmaceutics13060916

**Published:** 2021-06-21

**Authors:** Weiyi Liu, Greg Walker, Sally Price, Xiangdong Yang, Juan Li, Craig Bunt

**Affiliations:** 1 Faculty of Agriculture and Life Sciences, Lincoln University, Lincoln 7608, New Zealand; weiyi.liu@lincoln.ac.nz (W.L.); sally.price@lincoln.ac.nz (S.P.); 2 School of Pharmacy, University of Otago, Dunedin 9054, New Zealand; greg.walker@otago.ac.nz; 3 Institute of Agricultural Resources and Regional Planning, Chinese Academy of Agricultural Sciences/Key Laboratory of Plant Nutrition and Fertiliser, Ministry of Agriculture and Rural Affairs, Beijing 100081, China; yangxiangdong@caas.cn (X.Y.); lijuan02@caas.cn (J.L.)

**Keywords:** electrospinning, semi-permeable membrane, membrane characterization, controlled release, nanoparticles, release mechanisms

## Abstract

Electrospun nanofibers have been extensively studied for encapsulated drugs releasing from the inside of the fiber matrix, but have been barely looked at for their potential to control release as a semi-permeable membrane. This study investigated molecular transport behaviors across nanofiber membranes with different micro-structure sizes and compositions. Four types of membranes were made by 5% and 10% poly (ε-caprolactone) (PCL) solutions electro-spun with or without 50 nm calcium carbonate (CaCO_3_) nanoparticles. The membranes were tested for thickness, fiber diameter, pore size, porosity, tensile strength and elongation, contact angle of water and their impacts on molecular transport behaviors. The presence of the CaCO_3_ nanoparticles made the 5% membranes stronger and stiffer but the 10% membranes weaker and less stiff due to the different (covering or embedded) locations of the nanoparticles with the corresponding fibers. Solute transport studies using caffeine as the model drug found the 5% membranes further retarded release from the 10% membranes, regardless of only half the amount of material being used for synthesis. The addition of CaCO_3_ nanoparticles aided the water permeation process and accelerated initial transports. The difference in release profiles between 5% and 10% membranes suggests different release mechanisms, with membrane-permeability dominated release for 5% PCL membranes and solute-concentration-gradient dominated release for 10% PCL membranes.

## 1. Introduction

Electrospinning is a broadly used technique spinning either polymer solutions or melted polymers onto an earthed collector in a zone affected by strong electric fields. The membrane produced consists of numerous nano- or micro-scaled fibers interweaved and overlapped with each other to form fibrous and porous structures inside [1]. This technique has been shown to have many advantages such as producing nanofibers continuously and being easy to manipulate. It can be used with many types of polymers and suitable for various practical purposes. All these merits have made it popular over the last three decades. The membranes produced by electrospinning have been used in many fields including tissue engineering, filtration, biosensors, water-repellent clothing, energy generation, immobilization of enzymes, affinity membrane, cosmetics and biomedical applications [1,2,3].

Drug delivery is a sub-branch of biomedical application. A large number of studies have worked on incorporating drugs into polymer nanofibers and releasing the drugs from the inside of the nanofiber matrix. Loh et al. [4] created a thermoresponsive nanofiber formulation for controlled release of the protein bovine serum albumins (BSA) by electro-spinning a mixture of a poly (ester urethane)s solution with the addition of the protein. The release rate of the protein can be controlled by altering the temperature in release environments. Yohe et al. [5] incorporated 7-ethyl-10-hydroxycamptothecin (SN-38) into a super-hydrophobic nanofiber membrane (water contact angle > 150°) made by poly (ε-caprolactone) (PCL) and poly (glycerol monostearate-co-ε-caprolactone) and found that the air trapped in the membrane acted as a natural barrier, further retarding drug release from conventional hydrophobic membranes.

Although the research to incorporate drugs into fiber structures is well established, very few researchers have investigated controlling their release using nanofiber membranes as a physical barrier. To our knowledge, only three articles have published results in this area. Falde et al. [6] used the same super-hydrophobic nanofiber membrane made in [5] but employed it as a barrier layer to control release. It has been found the air trapped in the membrane functioned as good as when it was used in matrix systems to retard release. Scaffaro et al. [7] sandwiched a carvacrol (CRV) loaded polylactic acid (PLA) film with PLA nanofiber membranes of different thicknesses. Their results showed a diminished burst release of CRV with the increase of membrane thickness. The release of CRV across the PLA membranes has been found dominated by a diffusion mechanism. Fouling is a common phenomenon for microfiltration. It describes a situation where the flux of molecules is reduced during filtration due to the blockage of electrospun membrane pores caused by the interaction between foulants and the membrane [8,9]. Zhao et al. [10] found the fouling effect also existed in molecular transport processes. They investigated the impact of the fouling on the release of a protein BSA across polyacrylonitrile (PAN) nanofiber membranes using side-by-side diffusion chambers and observed a significantly lower diffusion coefficient produced for electrospun fibrous membranes than those for other fibrous media when the ratio of the protein size and pore size reached a certain value.

We obtained inspiration to control molecular release across nanofiber membranes from the above studies. If the thickness, hydrophobicity and molecular interaction of fibers and transporting molecules can be a tool to impact release, would the fiber diameter and pore size be another tool to manipulate the release? It has been well reported that the fiber diameter produced by electrospinning can be reduced by decreasing the polymer concentration used for synthesis process [11,12,13]. It was also reported in microfiltration that the flux of molecules can be altered by changing the microstructure of the nanofiber membranes such as the fiber diameter and porosity [14]. With these research tips, we conducted experiments on 5% and 10% PCL nanofiber membranes and hypothesized that the 5% membranes with thinner fibers and smaller pores would decrease release rates from the 10% membranes due to lower permeability. Nanoparticles (NPs) were another tool used in this study to manipulate the release rate. Many studies have found that by adding hydrophilic fillers, the water permeability of polymer membranes can be increased [15,16,17]. It was curious to understand whether the hydrophilic NPs can be used to accelerate the release rate of electrospun membranes. For this purpose, commercial calcium carbonate (CaCO_3_) NPs with a diameter of 50 nm were electro-spun with 5% and 10% PCL solutions to make nanofiber membranes containing hydrophilic fillers.

It has been found in this study that the NPs impacted the mechanical strength and elasticity of the membranes and made the 5% PCL membranes stronger and stiffer but the 10% PCL membranes weaker and less stiff. After eliminating the impacts of membrane thickness, hydrophobicity and the fouling phenomenon, it has been found that the 5% membranes can further retard release from the 10% membranes due to smaller pore size. The difference between accelerated and decelerated release styles appeared on 5% and 10% membranes proposed different working release mechanisms, with membrane-permeability dominated release for 5% membrane and solute-concentration-gradient dominated release for 10% membrane. The additions of CaCO_3_ NPs were not able to change the overall styles of the release but increased the initial release rate.

## 2. Materials and Methods

### 2.1. Materials

Poly (ɛ-caprolactone) (PCL) (MW 80,000) and caffeine (98.5% purity) were purchased from Sigma-Aldrich, Auckland, New Zealand. Dichloromethane (HPLC grade) was obtained from Fisher Scientific, Loughborough, UK. Methanol (laboratory-grade) was purchased from Thermo Fisher Scientific, Auckland, New Zealand. Calcium carbonate (CaCO_3_) nanoparticles with a diameter of 50 nm and a purity of 98% were acquired from US Research Nanomaterials, Houston, TX, USA.

### 2.2. Membrane Preparation by Electrospinning

**Figure 1:**
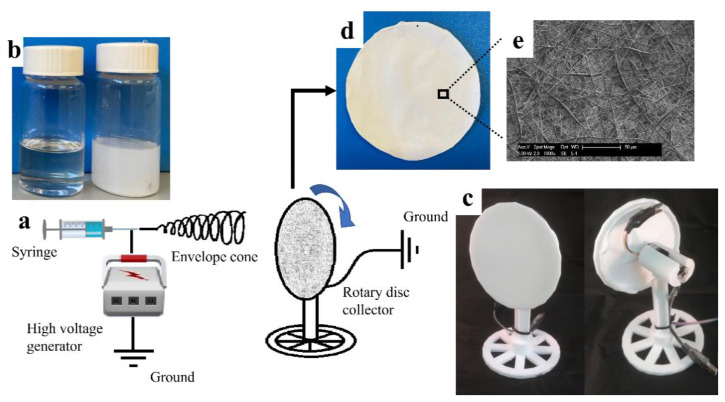
Schematic illustration of (**a**) the electrospinning process, (**b**) a 10% Poly (ɛ-caprolactone) (PCL) solution (left bottle) and a 10% PCL solution with calcium carbonate (CaCO_3_) nanoparticles (NPs) uniformly dispersed (right bottle) in DCM: Methanol = 3:1, (**c**) rotary disc collector used to collect nanofiber membranes, (**d**) a piece of 10% nanofiber membrane, (**e**) Scanning electron microscopy (SEM) image of a 10% membrane sample.

Poly (ɛ-caprolactone) nanofiber membranes were fabricated by adding 0.4 g PCL, 0.8 g PCL, 0.38 g PCL with 0.02 g CaCO_3_ nanoparticles and 0.76 g PCL with 0.04 g CaCO_3_ nanoparticles, respectively, into an 8 mL solvent consisting of dichloromethane and methanol in a ratio of 3:1 to make 5%, 10%, 5% with 5% CaCO_3_ NPs (to the weight of the solute), and 10% with 5% CaCO_3_ NPs (to the weight of the solute) membranes. Samples were stirred for 1 h until a homogenous solution or dispersion was obtained before being transferred to a 5 mL syringe (TERUMO^®^). A voltage of 20 kV supplied by a high voltage generator (DEL HVPS INST 230 30KV, Spellman^®^) with a solution feed rate of 4 mL h^−1^ and a flat end (23 gauge, TERUMO^®^) needle-to-collector distance of 20 cm was employed as the conditions for electro-spinning over a period of 1 h. All the samples were produced at room temperature with a humidity range from 40% to 60%. An earthed disc-shaped collector with a diameter of 10 cm, covered with aluminum foil (mono^®^) was rotated at 30 rpm during nanofiber collection. A set of images and photos illustrating the membrane preparation process is displayed in Figure 1.

### 2.3. Thickness Measurement

The thickness of nanofiber membranes was measured using micrometer calipers (mi004, Metalworking, Fuzhou, China). To minimize the compression impact of the measuring rods of the caliper, the membranes were folded twice before measurement. Three points being 1 cm, 2.5 cm and 4 cm away from the center of the circular membranes were measured. The thicknesses showed in this study were the averages of the measurements divided by 4. A detailed explanation of this method is available to view in the Supplementary File.

### 2.4. Fiber Morphology and Membrane Characterization

Nanofiber membrane samples were collected minimizing physical changes to its structure and stored at ambient room temperature. Scanning electron microscopy (SEM, Zeiss Sigma VP FEG/ Phillips XL30 FEG) was used to study the surface morphology of the membranes. The side of the membrane in contact with the air and collecting fibers in the spinning process was placed facing upward on a stub and coated with a 20 nm platinum layer before imaging with the SEM. Each sample was tested in 5000× and 20,000× magnifications for the overall morphology and the appearance of the CaCO_3_ NPs. Fiber diameter and pore size were measured using ImageJ (Fiji, National Institute of Mental Health, Bethesda, MD, US) on the SEM images. The average of 60 diameters and 100 successive pore areas were measured to obtain the values for fiber diameter and pore size. The mass of each 10 cm diameter samples was weighed and calculated with their thicknesses to obtain the membrane densities. The porosity of the membranes was calculated using the Equation (1) [18]:

(1) $$Porosity\;\left(\%\right)=\left(1-\frac{\rho }{\rho_{0}}\right)\times 100\%$$

where:

ρ = the density of electrospun PCL membrane;
$\rho_{0}$ = the density of bulk PCL polymer (1.14 g/cm^3^).

### 2.5. Tensile Strength and Elongation

A puncture test was carried out to measure the tensile properties of the nanofiber membranes. In the measurement, membrane samples were fixed in a ring clamp having a 2 cm diameter hole. The clamp loaded with the membrane samples was placed on the testing stage of a TA.XT plus Texture Analyzer (Stable Micro Systems) with the surface of the membranes facing up. A testing probe 5P/S and 5 kg load cell were used to punch the membranes in the hole from the top at a pre-test speed of 2 m ms^−1^ and test speed 0.5 m ms^−1^ until the samples were ruptured. The trigger force to start the measurement was 1 g. During the process, force and probe moving distance were recorded to draw the stress–strain curves of the membranes. Elastic property was assessed using the Young’s Modulus calculated from the slope of the linear elastic region of the curves. The equations to calculate these parameters are showed below [19,20]:

(2) Stress (MPa)=F/A

(3) $$A\;\left(m^{2}\right)=2rh$$

(4) $$Strain\;\left(\%\right)=\frac{\sqrt{r^{2}+d^{2}}-r}{r}\times 100\%$$

(5) $$Young^{\prime }s\;Modulus\;\left(\mathrm{MPa}\right)=Stress/Strain$$

where:

*F* = the force required to deform the membranes (N);
*A* = the sectional area of nanofiber membrane (m^2^);
*r* = the radius of the tested membranes mounted on the clumping ring (cm);
*h* = the thickness of the tested membranes (m);
*d* = the displacement of membranes being punctured (cm).

### 2.6. Water Contact Angle

Water contact angles were measured as described by Williamsa et al. [21] using the ImageJ (version 1.46, National Institute of Mental Health, Bethesda, MD, USA) plugin DropSnake (version 2.1, Biomedical Imaging Group, Lausanne, Switzerland). To obtain the angle, a 4 μL droplet of Reverse osmosis (RO) water was gently pipetted onto the nanofiber samples of which the size is large enough to ensure complete contact between the waterdrop and the membrane samples. A digital microscope (Microscope 2MP Digital 500× 8LED, Andonstar, Shenzhen, China) was used to capture the shape of the water drop. The original pictures were then edited into a perfectly horizontal and tidy appearance for the best performance of the measurement. The contact angles used in this study were the averages of left and right angles measured by DropSnake. A schematic diagram for the apparatus arrangement is available to view in the Supplementary File.

### 2.7. Solute Transport Experiment

Solute transport through the nanofiber membranes was assessed using a side-by-side diffusion chamber (CHM3, WORLD PRECISION INSTRUMENTS) consisting of two 1.2 mL chambers with a 12 mm orifice. Circular membrane samples (a diameter of 2.5 cm) of 5%, 5% with NPs, 10% and 10% with NPs were mounted between the “donor” and “receptor” chamber. RO water (19 mL) was added to each of the chamber columns followed by 1 mL of 0.8 mg/mL caffeine solution added to the “donor” side and 1 mL of RO water added to the “receptor” side to make up 20 mL in each column. Samples (100 µL) were collected from the top side of both columns after 0.25, 0.5, 1, 2, 3, 4, 5, 6, 23, 24, 25, 26, 27, 28, 29, 30, 47, 48 h and analyzed for caffeine using a UV/Vis Spectrophotometer at 287 nm (Multiskan GO, Thermo Scientific^®^, Vantaa, Finland). Absorbances were converted to caffeine concentration by using a standard curve of 0, 10, 20, 30 and 40 µg/mL caffeine prepared in 100 mL water. Fisher’s least significant difference (LSD) was used to analyze the pairwise differences among the solute concentrations in the “receptor” side for the four types of membranes after 6, 23, 30 and 48 h.

## 3. Results and Discussion

### 3.1. Nanofiber Morphologies

**Figure 2:**
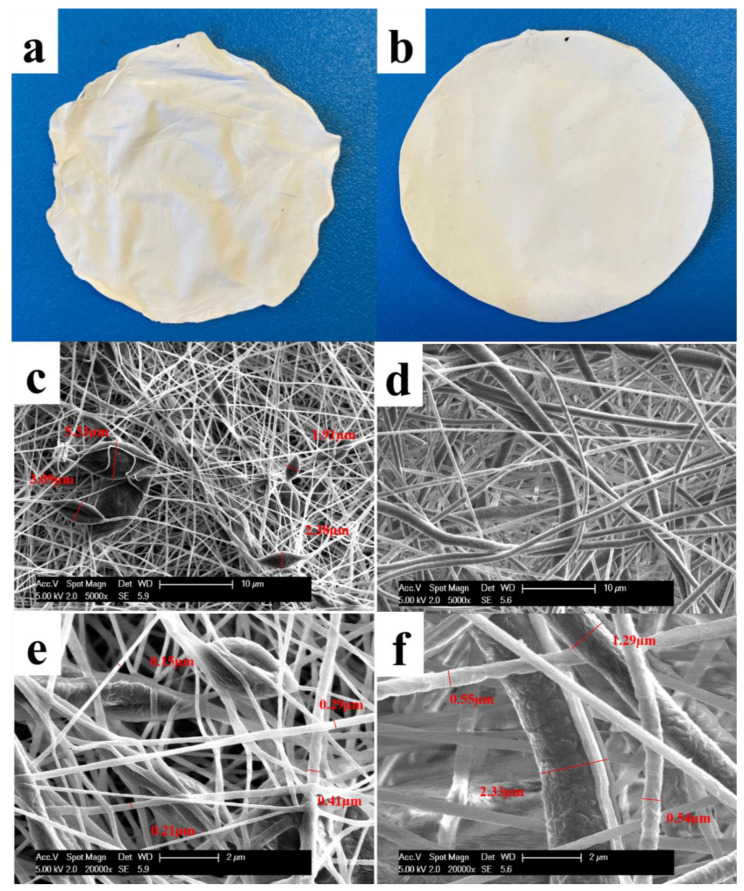
The macroscopic appearances of (**a**) a 5% PCL membrane and (**b**) a 10% PCL membrane. The SEM images of (**c**) 5% PCL nanofibers and micro-beads observed at 5000× magnification with some of the micro-beads measured for the widths, (**d**) 10% PCL nanofibers at 5000× magnification, (**e**) 5% PCL nanofibers and micro-beads at 20,000× magnification with some of the fibers measured for the diameters, (**f**) 10% PCL nanofibers at 20,000× magnification with some of the fibers measured for the diameters.

The appearances and fiber morphologies of 5% and 10% PCL membranes have been investigated in this study. Figure 2a,b illustrates the difference between the two membranes in appearance, with the 5% membrane exhibiting a softer and more flexible texture than the 10% membrane. The nanofiber morphologies of the two membranes were observed on SEM. The 5% PCL solution generated nano-scale fibers (diameter from 0.15 to 0.41 µm) with some micro-beads (waist width from 1.91 to 5.33 µm) connected in-line (Figure 2c,e). This is consistent with the literature reporting that polymer micro-beads were formed in electrospinning process when the concentration of the polymers in spun solutions decreased to a critical value [3,22]. The formation of the micro-beads is attributed to a break of entangled polymer chains into fragments before they reach the collector [23]. The 10% PCL solution, in contrast, created a fiber-only structure in micro-scale (diameter from 0.54 to 2.33 µm) (Figure 2d,f). The fibers produced by 5% PCL solution are narrower than those produced by 10% PCL solution. It has been well reported that the nanofibers produced by electrospinning enlarge with the increase of polymer concentration used for synthesis [11,12,13].

**Figure 3:**
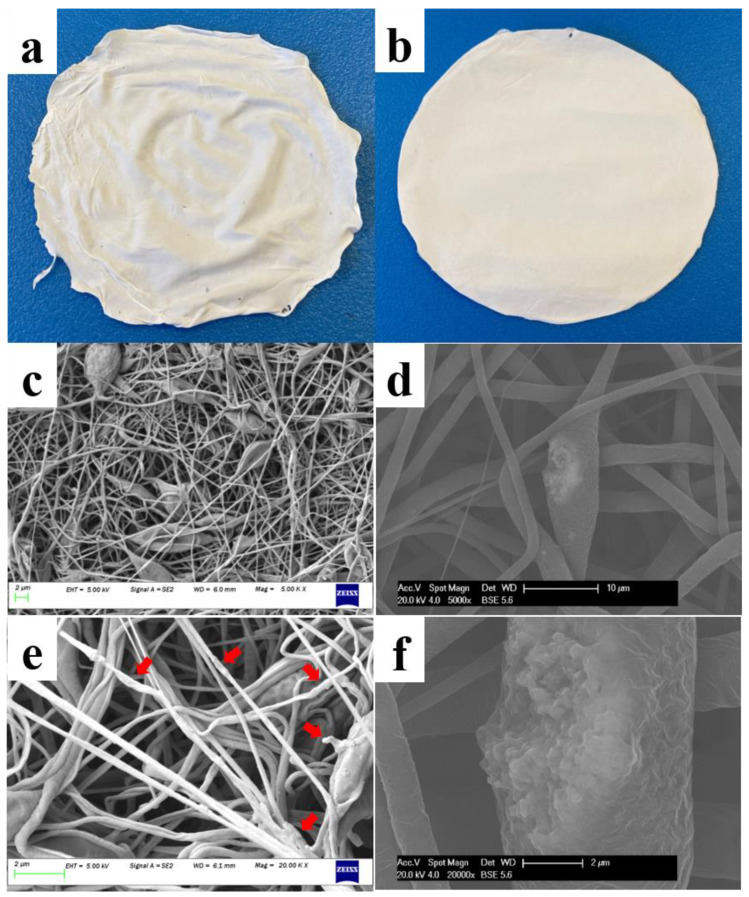
The macroscopic appearances of (**a**) a 5% PCL membrane spun with CaCO_3_ NPs and (**b**) a 10% PCL membrane spun with CaCO_3_ NPs. The SEM images of (**c**) 5% PCL nanofibers spun with CaCO_3_ NPs observed at 5000× magnification, (**d**) 10% PCL nanofibers spun with CaCO_3_ NPs observed at 5000× magnification, (**e**) 5% PCL nanofibers spun with CaCO_3_ NPs at 20,000× magnification, noticing the CaCO_3_ NPs covering on some fragments of the fiber surface, (**f**) a 10% PCL nanofiber embedded by a cluster of CaCO_3_ NPs at 20,000× magnification.

To evaluate the role of hydrophilic fillers, 5% CaCO_3_ NPs (to the weight of the solute) were electro-spun with 5% and 10% PCL solutions at the same spinning conditions. Calcium carbonate NPs were selected for this study as these particles have been widely proved to be feasible to incorporate with hydrophobic polymer fibers such as those made by PCL [24,25,26]. Figure 3a,b as well as Figure 2a,b shows that the addition of CaCO_3_ NPs had no impact on the macroscopic appearances of 5% and 10% membranes. A softer and more flexible texture has been found on the 5% membrane with NPs compared with that of the 10% membrane with NPs. After incorporated with the CaCO_3_ NPs, the 5% and 10% membranes showed no obvious changes in the fiber morphologies at 5000× magnification (Figure 3c,d compared with Figure 2c,d). A closer observation at 20,000×, however, revealed the different positions of the CaCO_3_ NPs incorporated with the corresponding fibers. As for the 5% fibers with NPs (Figure 3e), they had a rougher surface compared to the 5% fibers without NPs in Figure 2e. A similar rough surface was also created on the hydrophobic PAN nanofibers using the same NPs in the literature [27]. A number of fiber fragments with increased diameters (indicated by the red arrows in Figure 3e) emerged after the addition of the NPs, indicating that most of the NPs were covered on the surface of the 5% PCL nanofibers. Given that the size of the NPs (50 nm) is comparable to the diameters of the 5% fibers (averagely 230 nm showed in Table 1), the surface location of the NPs for 5% fibers is reasonable. The NPs incorporated in the 10% fibers, in contrast, aggregated to big clusters and comprised a part of the fibers (Figure 3f). A piece of literature using 12% PCL solution and a similar amount of CaCO_3_ NPs showed the same trend to our study with the NPs aggregating in the fibers [26]. A phase separation induced by the poor compatibilities of the hydrophobic fibers and hydrophilic NPs might be explained as the reason.

### 3.2. Membrane Characterization

The measurement results of thickness, fiber diameter and porosity properties of the 5% and 10% PCL membranes have been displayed in Table 1. The 10% PCL membranes had an average thickness of 110.2 µm and were 35.5% thicker than the 5% PCL membranes which had an average thickness of 81.3 µm. The average fiber diameter and pore size showed a much smaller micro-structure for the membrane made from 5% w/w PCL solution (0.23 µm for fiber diameter and 0.91 µm^2^ for pore size) compared to the membranes made from 10% w/w PCL solution (0.90 µm for fiber diameter and 9.19 µm^2^ for pore size). The increase in fiber diameter with higher polymer concentration is attributed to the higher stability of polymer jets and the stronger entanglements of the polymer chains induced by more viscous solutions [28]. Larger fibers form larger pore size due to the packing restriction during the fiber deposition process. A linear correlation between fiber diameter and pore size has been reported in the literature [29]. The pore size and fiber diameter of the 10% membrane measured in this study are consistent with the values reported in the literature using a similar solvent and the same concentration for synthesis [30,31]. The porosities of the 5% and 10% membranes were 83.0% and 72.5%, respectively. The data reported here are consistent with the common porosities of PCL membranes in literature which were in a range from 60% to 90% [32,33,34]. This difference in porosity indicates a greater void space in the 5% membrane compared to that in the 10% membrane. The result is consistent with the literature [35] showing that the porosity of electrospun scaffold reduced as the diameter of fibers increased.

### 3.3. Mechanical Properties of the Membranes

The tensile properties of the 5% and 10% PCL nanofiber membranes spun with or without CaCO_3_ NPs were measured in this study using a puncture test. The puncture test is a biaxial test designed to investigate the mechanical behaviors of two-dimensional (flat) materials under plane loading conditions [36]. Compared to a uniaxial tensile test, the puncture test simulated more closely the conditions associated with molecular transport driven by complex multi-loadings vertical to the plane of the membrane.

**Figure 4:**
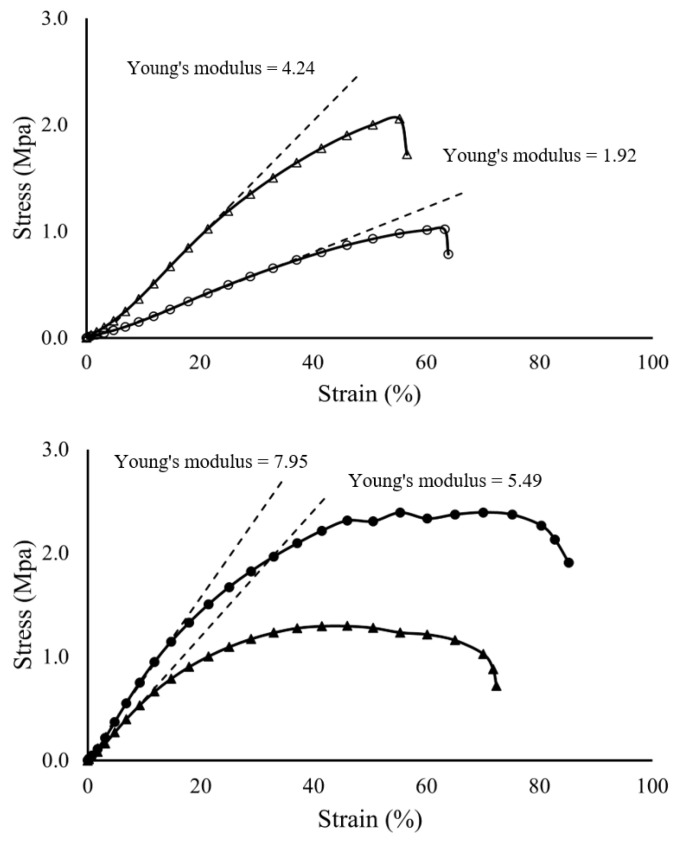
Stress–strain curves (*n* = 3) for: the 5% PCL membrane (○), 5% PCL membrane with CaCO_3_ NPs (△), 10% PCL membrane (●) and 10% PCL membrane with CaCO_3_ NPs (▲). The dashed lines are the trend of stress growth along with the increase of strain in the linear elastic region, indicating the Young’s modulus of each material.

Figure 4 showed that the puncture strength of the 5% PCL membrane is 1.02 MPa which is similar to the value of an 8% PCL membrane (1.2 MPa) reported in the literature [37]. The membrane spun from 5% PCL solution with 5% (to the weight of the solute) CaCO_3_ NPs was approximately twice as strong as the membrane without NPs (2.06 MPa compared with 1.02 MPa), indicating a stronger material created by the addition of the CaCO_3_ NPs. Likewise, Young’s modulus of the 5% membrane with CaCO_3_ NPs (4.24 MPa) was about twice that of the 5% membrane without NPs (1.92 MPa), which indicates that the addition of the NPs has made the membrane stiffer. The difference in membrane strength and elasticity (Young’s modulus) may be due to the CaCO_3_ NPs covered on the surface of the 5% nanofibers (Figure 3e) which has given additional resistance to the puncture test.

In contrast, the CaCO_3_ NPs has made the 10% membranes weaker and less stiff. The puncture strength of the 10% PCL membrane without NPs is 2.39 MPa while that of the 10% membrane with NPs is 1.30 MPa (Figure 4). This result is consistent with the tensile strength of a 10% PCL membrane reported in the literature being 2.7 MPa [38]. Young’s modulus for the 10% membrane (7.95 MPa) is a little higher than that of the 10% membrane with NPs (5.49 MPa), indicating a less stiff material has been made by the addition of the NPs. The possible reason for the difference in strength and elasticity (Young’s modulus) may be the NP clusters embedded in the fiber bodies in Figure 3d. The clusters might have made the fibers break more readily when being stretched due to the non-stretchable property of CaCO_3_ and the poor compatibility between the hydrophobic PCL and the hydrophilic CaCO_3_. The remained PCL nanofibers without NP clusters embedded kept the shape of the membrane for the rest of the analysis and produced a comparable elongation property to the membrane without NPs.

It has been reported that the tensile strength of electrospun nanofiber membranes is increased as the fiber diameter decreases due to the higher crystallinity and molecular orientation created in thinner fibers [39,40]. Our tensile results for the 5% and 10% membranes without NPs showed an opposite trend to the previous studies and this was attributed to the formation of micro-beads in the 5% PCL membrane. Other researchers who tested the strength of their membranes made using a series of polymer concentrations has given a similar result, showing a lower strength in the lower concentration membrane containing micro-beads [41].

### 3.4. Contact Angles

**Figure 5:**
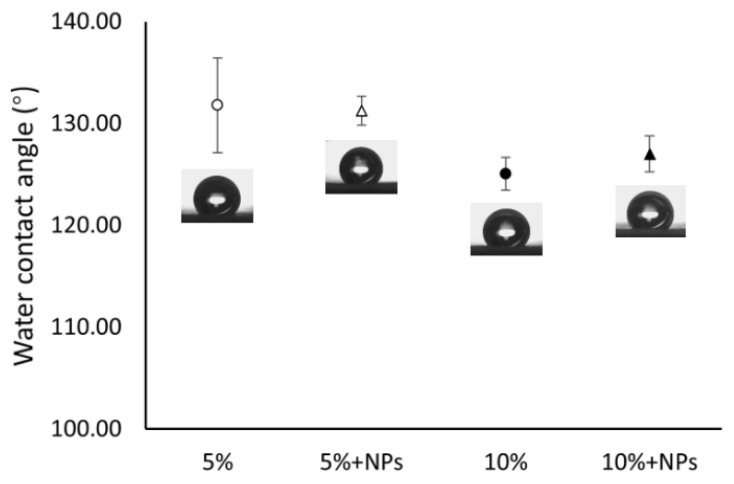
Contact angles of water droplets (*n* = 3) on membranes made using 5% and 10% PCL solution with or without NPs. The inserted figures under the error bars are the typical images of the drops on the corresponding nanofiber membranes.

The contact angles of waterdrops were measured for the 5%, 5% with NPs, 10% and 10% with NPs membranes. The angles were 131.8°, 131.2°, 125.1° and 126.5°, respectively (Figure 5). The contact angles are consistent with the angles reported in the literature [42,43,44] with a range from 112.4° to 132.0° for the spun PCL concentrations from 7.5% to 12%. A multiple comparison performed using the Tukey method has shown no statistical difference among the 4 contact angles (*p* > 0.05). This result is consistent with the literature where contact angles were reported to be independent of fiber diameters [45]. Unexpectedly the presence of CaCO_3_ NPs did not affect the hydrophobicity of the PCL membranes. A piece of literature showed that by adding an amount of CaCO_3_ NPs up to three times of the weight of spun PCL, there was only a limited contact angle increase [46]. Regarding the tiny amount of CaCO_3_ NPs added (5% to the weight of the solute) in this study, this result can be acceptable.

### 3.5. Solution Transport Capacity

**Figure 6:**
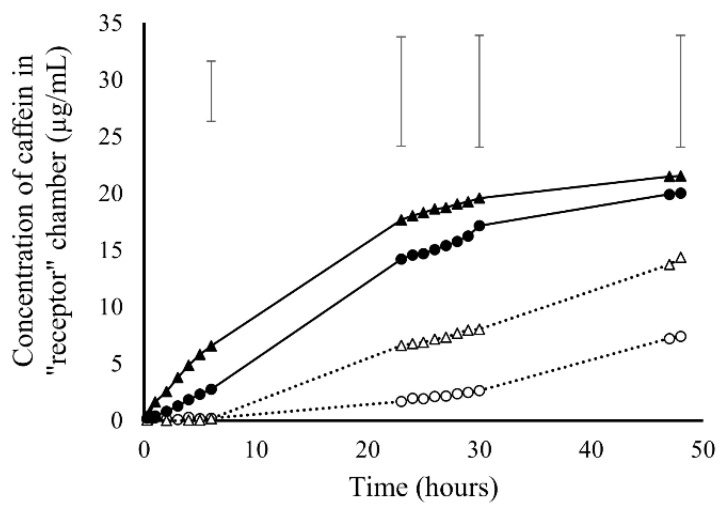
Caffeine transport behaviors (*n* = 3) through the 5% PCL membrane (○), 5% PCL membrane with CaCO_3_ NPs (△), 10% PCL membrane (●) and 10% PCL membrane with CaCO_3_ NPs (▲). The error bars are the LSDs for the concentrations of caffeine after 6, 23, 30 and 48 h at a 95% confidence level.

Solution transport behaviors across nanofiber membranes were investigated using caffeine as the model drug. Figure 6 shows the caffeine transport through the 5% and 10% membranes with or without CaCO_3_ NPs. The release profiles of the two concentrations differed from each other. Caffeine released through both 10% membranes with and without NPs from the beginning and showed a decreased release rate with time. In contrast, no caffeine was released during the initial 6 h for the 5% membranes with and without NPs, followed by an increased release rate with time. The addition of CaCO_3_ NPs was not able to change the overall release patterns but contributed to higher release rates in the initial 6 h for the 10% membranes and the initial 23 h for the 5% membranes. It has been reported that CaCO_3_ NPs can be a tool to increase water flux through polymer membranes by increasing their permeability [47]. In our study, the CaCO_3_ NPs aided the permeation of water through the membranes at an early period. The Fisher’s LSD error bars for the concentrations of caffeine after 6, 23, 30 and 48 h are showed above the solute transport graph. The analysis showed that a significant difference (at a 95% confidence level) in solute transport between the 5% membrane and the 10% membrane as well as between the 5% membrane and the 10% membrane with NPs emerged after 23 h and lasted to the end (48 h). This result indicates a unique formulation with a solute transport style different from the 10% membranes with and without NPs has been created using the 5% membrane.

**Figure 7:**
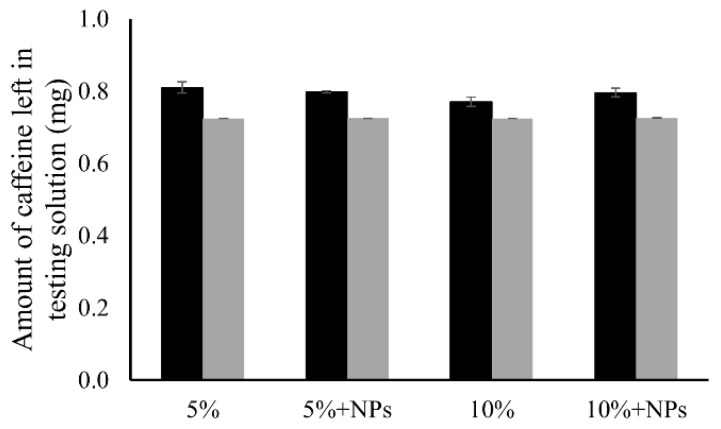
The measured amount (⯀) and calculated amount (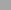) of caffeine left in the testing solutions after 48 h test for the 5% PCL membrane, 5% PCL membrane with NPs, 10% PCL membrane and 10% PCL membrane with NPs.

Caffeine was selected in this study as the model drug because it has a small molecular size and moderate compatibility to water and organic matter as a partially polar molecule [48,49]. By testing it at a low concentration (40 µg/mL), we are able to eliminate the impact of fouling effect. To confirm no fouling phenomenon existed in this transporting system, the calculated amount of caffeine left in the system after solution samples were extracted out was compared with the measured amount of caffeine left in both “donor” and “receptor” solutions. Figure 7 showed that the measured amounts of caffeine left in solutions were close to the calculated amounts, indicating that all the caffeine molecules dissolved in solution instead of being absorbed or immobilized on the membranes. The difference between measured and calculated amounts are attributed to the loss of water solvent by evaporation during the experimental period.

**Figure 8:**
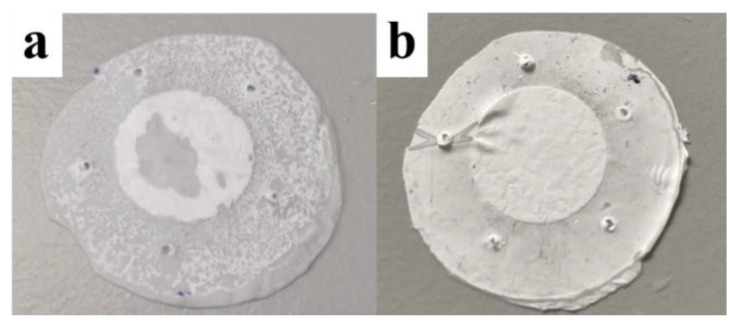
A typical example of a wetted area on the surface of (**a**) a 10% membrane and (**b**) a 5% membrane after 48 h solute transport experiments.

Based on the membrane characterization results in Section 3.2 and Section 3.4 where the impact of thickness and hydrophobicity on release behaviors has been removed, it is believed that the slower release rate of the 5% membranes is due to the 10-time smaller pore size compared to that of the 10% membranes (0.91 µm^2^ for the 5% membranes compared with 9.19 µm^2^ for the 10% membranes), which has given a higher transporting resistance and created a lower permeability for 5% membranes. A typical example for the wettability of the 5% and 10% membranes after 48 h transport experiments is shown in Figure 8 where a wetting zone can be seen on the surface of the 10% membrane (Figure 8a) while no wetting zone can be seen on the surface of the 5% membrane (Figure 8b).

**Figure 9:**
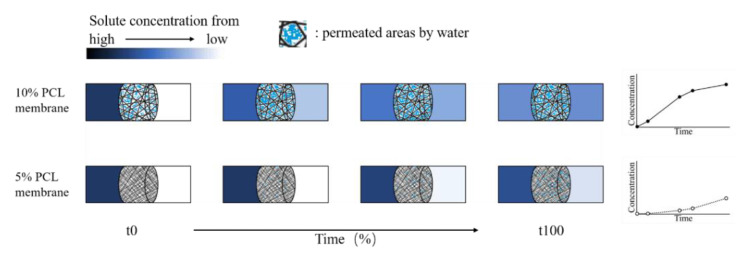
A demonstration of the release mechanisms proposed for the different release profiles for the 5% and 10% PCL membranes.

Based on all the results, we deduce that the difference in release profile between the 5% and 10% membranes resulted from different dominating release mechanisms. Figure 9 demonstrates what has happened on 5% and 10% membranes in our presumption. As for the 5% PCL membranes where wetting of the membranes is difficult, it is presumed that the release rate was increased with the expansion of the permeated areas. This type of release is mainly dominated by the permeability of nanofiber membranes. In contrast, the permeability of the 10% PCL membranes was so high that there was no significant impediment for the transport of the caffeine molecules. The molecules released according to the solute concentration gradient between the two sides of the membrane as the driving force. As the gradient decreased with time, the release rate dropped. This presumption is consistent with our observation in the molecular transport experiments where a positive relationship has been found between the concentration of caffeine in the “receptor” column and the area of wetted zone grown on membrane surface after 48 h. Unfortunately, the images of the wetted areas were not captured. Here, we propose a possible theory based on our understanding. In the same system as this experiment, as the pore size increased, the intrinsic permeability of the membrane increased leading to a transition of release rate from an accelerated style to a decelerated style with time. There should not be a critical value for the transition but a combined role with one driving factor (permeability or concentration gradient) dominating more than another based on the size of micro-structures. The accelerated and decelerated release styles changed with micro-structural dimensions might provide new opportunities for modulated or personalized pharmaceutical designs.

The membranes used in this study were produced under laboratory conditions with a productivity <1 g/h. Scale-up would be required for the industrial production of this application. A range of modifications to the conventional stationary one-needle electrospinning have been developed by using multi-needles, a spinneret with multi-holes, air-flow assistance, moving type spinnerets and/or needleless techniques. Productivity of the modified methods up to 450 g/h has been reported, which fulfils the requirement of industry [50]. Among the modifications, any approaches that might modify the final composition of the membranes in a detrimental manner should be excluded in order to ensure a controllable structure. This includes, but is not limited to, the use of the free surface method in which the open solution surface to generate multi-jets leads to a change of spun polymer concentration with time due to the escape of volatile solvents over the spinning period [51]. A layer-by-layer formulation is one of the promising designs for this application. Controlled release of carvacrol and SN-38 by sandwiching a drug-containing matrix between two pieces of electrospun membrane has been reported and highlighted the feasibility of the layer-by-layer design [6,7]. Other potential designs, such as using the membranes as a coating for drug tablets, need to be explored and evaluated in future research.

## 4. Conclusions

In this study, we investigated the role of microstructure and the addition of CaCO_3_ NPs on the molecular release across nanofiber membranes. It has been shown that both the 5% and 10% PCL nanofiber membranes can be used as a semi-permeable membrane to control the release of small and water-soluble molecule caffeine. The 5% membrane with nano-scaled fiber diameter and pore size showed a lower release rate than the 10% membrane with micro-scaled diameter and pore size. A difference in release profiles of the two membranes indicates different dominating release mechanisms, with membrane-permeability controlled release for 5% membranes resulting in accelerated release rates with time and solute-concentration-gradient controlled release for 10% membrane resulting in decelerated release rates with time. A theory has been proposed by this study that the release profile of solute transporting across nanofiber membranes can be manipulated by altering the size of the microstructure of the nanofiber membranes. It has also been found that CaCO_3_ NPs as a hydrophilic filler for the formulations can be used to enhance the permeability of the electrospun membranes and accelerate the initial release of the solute. The NPs, however, impact the mechanical strength and elasticity of the membranes and produced a stronger, stiffer 5% PCL membrane and a weaker, less stiff 10% PCL membrane than their counterparts without the NPs. The reduction in structural dimensions and the addition of hydrophilic NPs might be a tool to optimize electrospun membranes for particular uses.

## Figures and Tables

**Table 1:** Physical dimensions of poly (ɛ-caprolactone) nanofiber membranes.

Samples	Thickness (µm)	Fiber Diameter (µm)	Pore Size (µm^2^)	Porosity (%)
5% PCL	81.3 ± 6.3	0.23 ± 0.01	0.91 ± 0.11	83.0 ± 0.6
10% PCL	110.2 ± 5.9	0.90 ± 0.06	9.19 ± 0.89	72.5 ± 0.5

## Data Availability

The data presented in this study are openly available in Lincoln University at https://doi.org/10.25400/lincolnuninz.14569953.v1 (accessed on 15 May 2021).

## Supplementary Materials

The explanations for the measurement methods of membrane thickness and water contact angle are available online at https://www.mdpi.com/article/10.3390/pharmaceutics13060916/s1, Figure S1: A photo demonstration of membrane thickness measurement, Figure S2: The schematic diagram of contact angle measurement.
