# Supplementary material for: Electrospun Membranes as a Porous Barrier for Molecular Transport: Membrane Characterization and Release Assessment
Source: Pharmaceutics. 2021 Jun 21;13(6):916. doi: 10.3390/pharmaceutics13060916 (PMC8235673; doi:10.3390/pharmaceutics13060916)
Supplement: Supplementary file 1 [file pharmaceutics-13-00916-s001.zip › pharmaceutics-1243344-supplementary.pdf]

pharmaceutics

Supplementary Materials: Electrospun Membranes as a Porous Barrier for Molecular Transport: Membrane Characterization and Release Assessment

Weiyi Liu, Greg Walker, Sally Price, Xiangdong Yang, Juan Li and Craig Bunt

1. Thickness Measurement

Thickness measurements for triplicate samples of each treatment were conducted using a micrometre calliper (mi004, Metalworking). It has been found in pre-trails that the measuring rods of the micrometre calliper compressed the surface of nanofiber membranes and resulted in lower thickness values. To minimise the impact of the rods, the electro-spun circular membrane samples with a radius of 5 cm were folded twice along the vertical and horizontal diameters before the measurement. Three measuring points were designed 1 cm, 2.5 cm and 4 cm away from the centre of the circle. In the cases that the nanofiber membrane shrank after taken off from the collector, the measuring points were moved proportionally according to the diameter of the circular sample. The thicknesses of the membranes are calculated from the average of the three measurements divided by 4. A procedure illustration has been shown in Figure S1 to clarify the process.

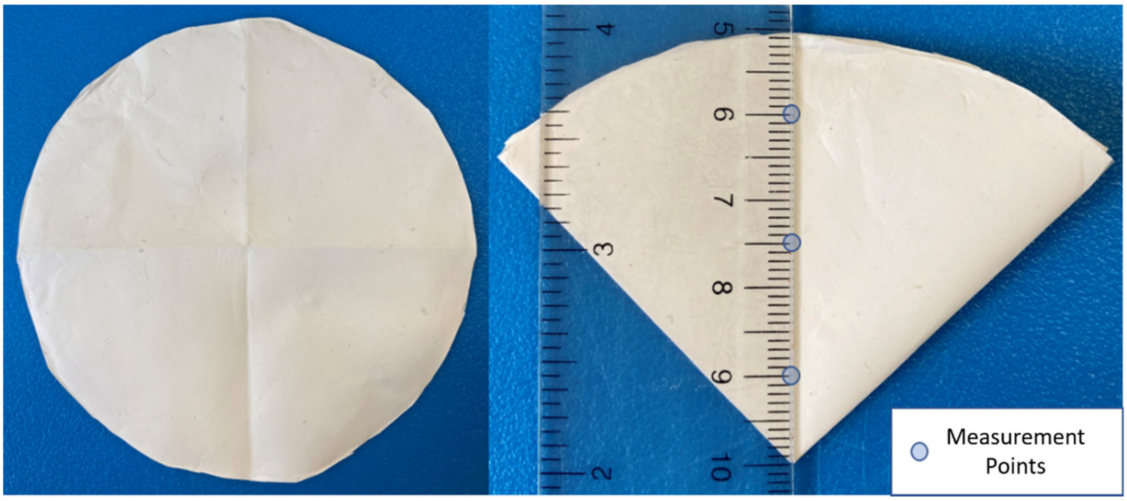

Figure S1. A photo demonstration of membrane thickness measurement. A piece of as-spun membrane sample (left) and a folded sample ready for measurement (right). The circular marks on the folded sample are the measuring points being 1 cm, 2.5 cm and 4 cm away from the centre of the membrane circles.

2. Water Contact Angle Measurement

Water contact angles in this study were measured using the ImageJ (version 1.46, National Institute of Mental Health, USA) plugin DropSnake (version 2.1, Biomedical Imaging Group, Switzerland). To avoid the impact of gravity on the shape of droplets, small droplets made by 4 µl RO water were employed throughout the experiments. A little piece of nanofiber membrane having the side receiving nanofibers in fabrication process facing upward was fixed on a sample stand which was adjustable horizontally in X and Y directions and vertically in Z direction. The 4 µL droplet was gently pipetted onto the membrane sample. A light source after being softened by paper tissue was gathered to the droplet area by a light barrier plate with a hole to enhance the contrast of the droplet to the environment. A digital microscope was placed in an opposite direction of the light source with the droplet in the middle to capture the shape of the droplet in shadow. The microscope was then adjusted in zoom until a clear image of the droplet was obtained.

The as-taken images were edited into a perfectly horizontal and tidy appearance for the best measurement performance of DropSnake. Figure S2 is the schematic diagram of the apparatus arrangement and a water droplet appearance during measurement.

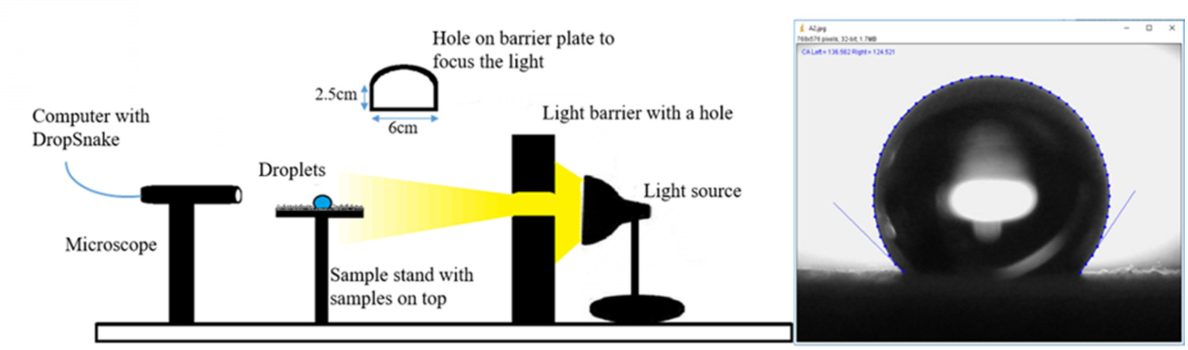

Figure S2. The schematic diagram of contact angle measurement. The layout of the water contact angle apparatus (left) and an image of a 4 µL water droplet in measurement (right).
